# Activation-Dependent Rapid Postsynaptic Clustering of Glycine Receptors in Mature Spinal Cord Neurons

**DOI:** 10.1523/ENEURO.0194-16.2017

**Published:** 2017-02-06

**Authors:** Yoshihisa Nakahata, Kei Eto, Hideji Murakoshi, Miho Watanabe, Toshihiko Kuriu, Hiromi Hirata, Andrew J. Moorhouse, Hitoshi Ishibashi, Junichi Nabekura

**Affiliations:** 1 Division of Homeostatic Development, Department of Developmental Physiology, National Institute for Physiological Sciences, Okazaki 444-8585, Japan; 2 Department of Physiological Sciences, The Graduate University for Advanced Studies (SOKENDAI), Okazaki 444-8585, Japan; 3 Supportive Center for Brain Research, National Institute for Physiological Science, Okazaki 444-8585, Japan; 4 PRESTO, Japan Science and Technology Agency (JST), Kawaguchi 332-0012, Japan; 5 Department of Neurophysiology, Hamamatsu University School of Medicine, Hamamatsu 431-3192, Japan; 6 Department of Neurophysiology, Kagawa School of Pharmaceutical Sciences, Tokushima Bunri University, Tokushima 769-2193, Japan; 7 Division of Molecular and Developmental Biology, National Institute of Genetics, Mishima 411-8540, Japan; 8 Department of Genetics, The Graduate University for Advanced Studies (SOKENDAI), Mishima 411-8540, Japan; 9 Department of Chemistry and Biological Science, Graduate School of Science and Engineering, Aoyama Gakuin University, Sagamihara 252-5258, Japan; 10 Department of Physiology, School of Medical Sciences, University of New South Wales, Sydney 2052, Australia; 11 Department of Physiology, Kitasato University School of Allied Health Sciences, Sagamihara 252-0373, Japan; 12 CREST, Japan Science and Technology Agency (JST), Kawaguchi 332-0012, Japan

**Keywords:** gephyrin, glycine receptor (GlyR), inhibitory synaptic plasticity, mature neuron, spinal cord, synaptic clustering

## Abstract

Inhibitory synapses are established during development but continue to be generated and modulated in strength in the mature nervous system. In the spinal cord and brainstem, presynaptically released inhibitory neurotransmitter dominantly switches from GABA to glycine during normal development *in vivo*. While presynaptic mechanisms of the shift of inhibitory neurotransmission are well investigated, the contribution of postsynaptic neurotransmitter receptors to this shift is not fully elucidated. Synaptic clustering of glycine receptors (GlyRs) is regulated by activation-dependent depolarization in early development. However, GlyR activation induces hyperpolarization after the first postnatal week, and little is known whether and how presynaptically released glycine regulates postsynaptic receptors in a depolarization-independent manner in mature developmental stage. Here we developed spinal cord neuronal culture of rodents using chronic strychnine application to investigate whether initial activation of GlyRs in mature stage could change postsynaptic localization of GlyRs. Immunocytochemical analyses demonstrate that chronic blockade of GlyR activation until mature developmental stage resulted in smaller clusters of postsynaptic GlyRs that could be enlarged upon receptor activation for 1 h in the mature stage. Furthermore, live cell-imaging techniques show that GlyR activation decreases its lateral diffusion at synapses, and this phenomenon is dependent on PKC, but neither Ca^2+^ nor CaMKII activity. These results suggest that the GlyR activation can regulate receptor diffusion and cluster size at inhibitory synapses in mature stage, providing not only new insights into the postsynaptic mechanism of shifting inhibitory neurotransmission but also the inhibitory synaptic plasticity in mature nervous system.

## Significance Statement

Clustering of postsynaptic glycine receptors (GlyRs) is critical for the developmental shift from GABAergic to glycinergic inhibitory neurotransmission in the spinal cord and brainstem. Synaptic GlyR localization is mediated by the receptor activation and followed by depolarization-dependent Ca^2+^ influx in the immature stage. However, little is known whether and how presynaptically released glycine enhances postsynaptic GlyR clustering in the mature stage when glycinergic transmission is upregulated without depolarization. Here we report postsynaptic GlyR clustering induced by the receptor activation in mature neurons. Furthermore, postsynaptic stabilization of laterally diffusive GlyRs is mediated in Ca^2+^-independent PKC activity at sites expressing gephyrin, a postsynaptic scaffolding protein. Our results provide new insights into homeostatic receptor dynamics underlying activation-dependent modulation of inhibitory synaptic strength in the mature nervous system.

## Introduction

GABA and glycine mediate fast inhibitory neurotransmission in the mature nervous system by activating postsynaptic GABA_A_ and glycine receptors, respectively, to reduce neuronal excitability via postsynaptic hyperpolarization and membrane shunting. The inhibitory neurotransmissions in the adult brainstem and spinal cord are predominantly mediated by glycine and play essential roles for the auditory system and the ascending nociceptive pathways (Sanes et al., 1998; Lynch and Callister, 2006). Interestingly, the predominant neurotransmission during the early developmental stage is GABAergic and developmentally shifts to glycinergic in these synapses (Kotak et al., 1998; Gao et al., 2001; Russier et al., 2002). This unique developmental feature is primarily regulated by switching the proportions of GABA and glycine in presynaptic vesicles (Nabekura et al., 2004). In the presynaptic terminals, vesicular inhibitory amino acid transporter [VIAAT (or VGAT)] uptakes both GABA and glycine with different binding affinity (McIntire et al., 1997); thus, the relative proportions of presynaptic glycine and GABA concentrations determine the contents of released inhibitory neurotransmitters (Ishibashi et al., 2013). Therefore, with developmental reductions of glutamic acid decarboxylases, GABA-synthesizing enzymes regulate the switching of presynaptically released inhibitory neurotransmitters (Ma et al., 1994; Nabekura et al., 2004).

Although the presynaptic mechanism underlying the developmental shift of inhibitory neurotransmission has been well investigated, it is still has not been elucidated whether and how postsynapses contribute to the shift of inhibitory neurotransmission. The postsynaptic localization of appropriate neurotransmitter receptors is primarily essential for the effective neurotransmission, and two scenarios in postsynaptic receptor localization are postulated for the developmental shift of neurotransmission. One scenario is that both GABA_A_ receptors and glycine receptors (GlyRs) are preexisting and stable; therefore, the developmental shift of neurotransmission is purely attributable to the proportions of presynaptically released GABA/glycine. The other scenario is that postsynaptic receptor localization is actively modulated in correspondence with developmental switching of released neurotransmitters. The number of receptors clustering postsynaptically is determined first by trafficking of the receptors between the cytoplasm and the plasma membrane at extrasynaptic sites (exocytosis and endocytosis), and, second, by the lateral diffusion of receptors in the plasma membrane and their stabilization at synaptic sites (Jacob et al., 2008). Extensive studies on excitatory synapses indicate that neurotransmission-dependent stabilization of laterally diffusing postsynaptic receptors may be a principal determinant of excitatory synaptic potentiation (Huganir and Nicoll, 2013). Similarly, the inactivation of postsynaptic GlyRs suppressed formation of the receptor clusters during early development in spinal cord neurons (Kirsch and Betz, 1998; Lévi et al., 1998), indicating that the neurotransmitter itself promotes receptor clustering. Thus, the neurotransmission-dependent clustering of GlyRs was explained by the GlyR-mediated depolarization and subsequent Ca^2+^ influx and CaMKII activation that occur when GlyRs are activated in early development (Kirsch and Betz, 1998; Yamanaka et al., 2013).

GABA_A_ receptor- or GlyR-mediated neurotransmissions developmentally shift from depolarizing to hyperpolarizing responses (D–H shift) after the first postnatal week with decreasing intracellular Cl^−^ concentration (Kakazu et al., 1999; Ben-Ari, 2002). However, the expression of GlyRα1, the mature-type subunit constructs the heteromeric GlyR, which localizes at postsynapses, and increases in parallel with the upregulated presynaptic glycine releases, and this persists after the D–H shift up to at least the third postnatal week (Friauf et al., 1997; Korada and Schwartz, 1999). Therefore, the activation-induced depolarization model in early development may be insufficient to explain the postsynaptic clustering of GlyRs in the mature stage, and we hypothesize that the postsynaptic localization of GlyRs are regulated by presynaptically released glycine in a depolarization-independent manner in mature stage. To address this question, we generated a spinal neuron culture model to distinguish and mimic the initial activation events of GlyRs after the D–H shift. In this model, we chronically prevented activation of GlyRs by strychnine, a competitive GlyR antagonist treatment, during the developmental period. Then GlyRs are activated after the D–H shift by strychnine removal. Thus, this model enables us to analyze the effects of GlyR activation on the spatiotemporal dynamics of postsynaptic GlyRs in mature neurons. In contrast to the current depolarization-dependent model of GlyR clustering, we found that the activation of GlyRs in more mature neurons could still elicit changes in diffusion and increases in the postsynaptic GlyR clusters.

## Materials and Methods

All procedures performed in this study were approved by the Okazaki Institutional Animal Care and Use Committee and were conducted in accordance with guidelines defined by the National Institute of Natural Sciences. All efforts were made to minimize the suffering and number of animals used in this study.

### Primary spinal cord culture

Dissociated primary cultures of spinal cord neurons were prepared as described previously (Ishibashi et al., 2013). Wild-type C57BL/6 mice (Japan SLC) were used for electrophysiology and experiments with DNA transfection, VGAT-Venus transgenic C57BL/6 mice (Wang et al., 2009) were used for quantum dot (QD) single-particle tracking (SPT) and wild-type Wistar rats (Japan SLC) were used for immunocytochemistry and immunoblotting. Briefly, embryos [embryonic day 13 (E13) to E15] of both sexes were isolated from pregnant animals under isoflurane anesthesia and were immediately placed into ice-cold Leibovitz’s L-15 medium (Life Technologies). Spinal cords were quickly removed and incubated in HEPES-buffered DMEM containing papain (24 µl/ml; Worthington) for 20 min at 32ºC. Individual cells were dissociated by repeated gentle pipette trituration in Neurobasal medium containing DNase and l-glutamate. Neurons were collected by centrifugation at 8000 rpm for 1 min, and the supernatant was exchanged with fresh Neurobasal medium containing B27 supplement (50×), Glutamax (Life Technologies), penicillin 100 U/ml, and streptomycin 100 µg/ml. Cells were plated on polyethylenimine-coated coverslips, either 18 mm diameter or 8 × 8 mm (Matsunami) at a density of 1.0–1.6 × 10^4^ cells/cm^2^, and were incubated at 37°C and 5% CO_2_ for 14–42 d before the experiments.

Three experimental groups were typically used (see Fig. 2*A*). The first group [chronic strychnine (cSTR)] was chronically treated with strychnine (1 µm) to block GlyR activation from the first day of culture through to when experiments were performed after at least 2 weeks in culture, when neurons and their intracellular Cl^−^ concentrations were equivalent to mature neurons. The second group was similarly treated with chronic administration of strychnine, but strychnine was then washed out 1 h prior to each experiment (WASH group). Hence, neurons in this group had GlyRs activated by endogenous synaptic activity for 1 h. The third group [control (Ctrl)] was treated with vehicle (Neurobasal culture medium, the solvent of strychnine) from the first day of culture plating, resulting in normal activation of GlyRs throughout development from endogenous synaptic activity.

### DNA Constructs and transfection

The coding sequence of superecliptic pHluorin (SEP; a gift from Dr. G. Miesenböck, University of Oxford, Oxford, UK) was fused at the extracellular domain of the mouse GlyRα1 subunit, just following the signal peptide sequence (pCAG-SEP-GlyRα1). The mCherry–gephyrin (GPHN) construct was constructed from the rat gephyrin-P1 splice variant 1, which was labeled at its N terminus with mCherry (Kuriu et al., 2012). Cells were transfected 13–15 d *in vitro* (DIV) and imaged within 48 h after transfections. Transfections were performed by incubating cell in Lipofectamine 2000 (Life Technologies) in Neurobasal medium lacking penicillin-streptomycin for 6–8 h.

### Reagents

Drugs used in the present study were 5 µm dl-2-amino-7-phosphonovalerate (dl-APV), 5 µm 6-cyano-7-nitroquinoxaline-2,3-dione (CNQX), 1 µm 6-imino-3-(4-methoxyphenyl)-1(6*H*)-pyridazinebutanoic acid (SR95531), 1 µm strychnine, 20–40 μg/ml gramicidin D, 100 µm or 1M glycine, 5 µg/ml brefeldin A (BFA), 1 µm ginkgolide B, 0.5 µm mecamylamine hydrochloride, 10 µm BAPTA-AM, 30 µm KN-62, 10 µm Rp-adenosine 3, and 1 µm ginkgolide monophosphorothioate triethylammonium salt (all from Sigma-Aldrich Japan); 50 nm GF 109203X (GFX; Enzo Life Sciences); and 0.3 µm tetrodotoxin (TTX; Latoxan). Drug-containing solutions were locally applied via a Y-tube perfusion system (Murase et al., 1989) in electrophysiological recordings and via bath application in imaging experiments.

### Solutions

The standard extracellular solution for imaging and electrophysiological experiments contained the following (in mm): 148 NaCl, 5 KCl, 1 MgCl_2_, 2 CaCl_2_, 10 glucose, and 10 HEPES, and adjusted to pH 7.4 with Tris-OH. In all experiments, the standard extracellular solution contained the (in μm) 5 CNQX, 5 dl-APV, 1 SR95531, and 0.3 TTX, concentrations that were sufficient to fully inhibit spontaneous excitatory and GABAergic postsynaptic currents, and action potentials. Additionally, 1 μm strychnine was constantly present in the cSTR group and was present in the WASH group until 1 h before experiments. QD binding buffer contained the following (in mm): 2.62 sodium tetraborate decahydrate, 39.5 boric acid, and 2% (w/v) bovine serum albumin and adjusted to pH 7.4 with 1 m HCl or 1 m NaOH. In electrophysiological experiments, the internal pipette solution for the perforated patch-clamp configuration contained the following (in mm): 90 KCl, 60 K-methanesulfonate, and 10 HEPES. The pipette solution for conventional whole-cell configuration contained the following (in mm): 100 K-methanesulfonate, 8 NaCl, 30 KCl, 1 MgCl_2_, 2 EGTA, 4 Mg-ATP, 5 QX-314-Cl, and 10 HEPES. All pipette solutions were adjusted to pH 7.3 with Tris-OH. In a perforated patch-clamp configuration, gramicidin D was initially dissolved in DMSO at 10 mg/ml and then diluted to a final concentration of 20–40 µg/ml in the pipette solution. The gramicidin was prepared just before use.

### Electrophysiological measurements

Cultured neurons were observed under phase contrast on an inverted microscope (model IX70, Olympus). Ionic currents were measured with a Multiclamp 700B patch-clamp amplifier (Molecular Devices) and were recorded with a sampling frequency of 20 kHz after low-pass filtering at 5 kHz. Currents were recorded, and voltage protocols were applied, using pClamp 10.2 software (Molecular Devices; RRID: SCR_011323). The resistance between the patch pipette filled with the internal solution and the reference electrode in the standard external solution was 6–8 MΩ. The neurons were voltage clamped at a holding potential of −53 mV in gramicidin-perforated patch experiments and −70 mV in the conventional whole-cell patch-clamp configurations.

The reversal potentials of glycine-induced currents (*E*_gly_) were recorded using gramicidin-perorated patch configurations to maintain intracellular Cl^−^ concentrations intact (Ebihara et al., 1995). Voltage-ramps from –113 to +37 mV, with duration of 1.6 s, were applied to the neuron before and during glycine application. Voltage protocols were generated by a software-driven digital-to-analog converter (Digidata 1322A, Molecular Devices). Corrections for the liquid junction potentials (–3.4 mV) were applied to the data in gramicidin-perforated recordings. Current–voltage (*I–V*) relationships were measured by subtracting current values during the control from those during glycine application, and the *E*_gly_ was quantified as the reversal potential of this control subtracted current.

Spontaneous mIPSCs were recorded using a conventional whole-cell patch-clamp configuration. To measure mIPSCs, in the cSTR group the bath solution including strychnine was completely exchanged with the standard recording solution lacking strychnine immediately before recording. In the control group, the bath solution was exchanged to a recording solution that included 1 µm strychnine for 30 s and then was exchanged again to the standard solution lacking strychnine in order to exclude the effect of any residual strychnine between the control and chronic strychnine treatment groups. Recordings started within 10 min after strychnine washout and lasted for 60 min. mIPSCs were confirmed by application of 1 µm strychnine after each recording. All experiments were performed at room temperature (21 − 24ºC). Recordings were discontinued if the access resistance changed >30%.

Spontaneous mIPSCs were analyzed using the template search tool in Clampfit 10.2 (Molecular Devices). The template was created from 100 responses and automatically detected events. Amplitude threshold values were set at threefold of SD values of the baseline noise amplitude. False-positive results were rejected by visual inspection. Over 200 events were detected and statistically analyzed at each time point ± 2.5 min from each neuron. Decay time constants were calculated by fitting a double-exponential function to the mIPSC decay from the time period corresponding to that between 90% and 10% of the peak mIPSC amplitude.

### Immunostaining, image acquisition, and quantitative analysis

Cultured rat spinal cord neurons were fixed with paraformaldehyde (PFA; 4% w/v) in PBS and then permeabilized with Triton X-100 (0.1% v/v). Nonspecific antibody-binding sites were blocked with PBS containing normal goat serum (2% v/v) and bovine serum albumin (1% w/v). Cells were double labeled using the following primary antibodies: polyclonal rabbit anti-VIAAT (1:500; Synaptic Systems; RRID: AB_887871), polyclonal guinea pig anti-VIAAT (1:1000; catalog #131-004, Synaptic Systems; RRID: AB_887873), monoclonal mouse anti-glycine receptor α1 (mAb2b; 1:200; Synaptic Systems 146-111; RRID: AB_887723), and polyclonal rabbit anti-gephyrin (1:1000; catalog #147-003, Synaptic Systems; RRID: AB_887718) overnight at 4°C, followed by incubation with fluorescence-conjugated secondary antibodies (Alexa Fluor 488, RRID: AB_2534117 or AB_2534069, 1:1000; Alexa Fluor 594, RRID: AB_2534095, 1:1000; Alexa Fluor 633, RRID: AB_2535731, 1:1000; Life Technologies) for 30 min at room temperature. Coverslips were mounted in PermaFluor aqueous mounting medium (Thermo Shandon). Fluorescence images were acquired with a confocal laser-scanning microscope [Plan Apo-VC 100× H, numerical aperture (NA) 1.4; model A1, Nikon]. Pixel size and focus steps were 0.1 and 0.37 µm, respectively, with images of 1024 × 1024 pixels.

The number and areas of puncta were measured using MetaMorph 7.7 (Molecular Devices) as described previously (Kuriu et al., 2012), with slight modifications. Maximal intensity projection images were prepared for each image stack, and these projection images were filtered using a 3 × 3 pixel low-pass filter to remove noise. Images were converted to binary images using an intensity threshold to determine the outline of the punctate signals. After applying built-in erosion and dilation processing to remove single-pixel noises, the numbers and areas of fluorescence signals were automatically counted from binary images using the integrated morphometry analysis function. Colocalization of immunostained presynaptic marker proteins (VIAAT) and postsynaptic GlyRs was determined using Wright Cell Imaging Facility ImageJ software (RRID: SCR_008488) based on the overlap coefficient of Manders et al. (1993) after automated background subtraction to avoid user bias in setting analysis parameters. A colocalization coefficient value of 0 indicates nonoverlapping immunostained proteins, while a coefficient of 1 corresponds to 100% colocalization. The coefficient indicates the proportion of the GlyR or GPHN signals coincident with the VIAAT signal intensity. Results were shown as the ratio of the summed intensities of pixels from the GlyR or GPHN for which the intensity of VIAAT was above zero.

### Cell surface biotinylation and immunoblotting

Surface biotinylation experiments were performed using a cell surface protein biotinylation and purification kit (ThermoFisher Scientific) according to the manufacturer protocol. Briefly, cultured spinal cord neurons from four individual cultures (19–33 DIV) were washed with ice-cold PBS and then labeled with 0.25 mg/ml sulfo-NHS-SS-biotin for 30 min at 4°C before washing with PBS supplemented with quenching solution to remove excess biotin. The cell lysates were centrifuged (10,000 × *g*, for 2 min), the supernatant was isolated with NeutrAvidin gel, and the bound proteins were then eluted with SDS sample buffer (62.5 mm Tris-HCl, pH 6.8, 1% SDS, and 10% glycerol) and were analyzed by SDS-PAGE and Western blotting.

Proteins were separated using 7.5% acrylamide gels by SDS-PAGE. The gels were transferred to an Immobilon-P membrane (Millipore). The blots were blocked in 1% bovine serum albumin and incubated overnight with primary antibody at 4°C. They were then incubated with horseradish peroxidase-conjugated secondary antibody (GE Healthcare UK) for 1 h at room temperature. Enhanced chemiluminescence (ECL; GE Healthcare) exposure on instant film and an ECL mini-camera luminometer were used to visualize labeled protein. The following primary antibodies were used: anti-pan GlyRs (1:1000; mAb4a, Synaptic Systems; RRID: AB_887722) and anti-actin (1:10,000; Sigma-Aldrich- RRID: AB_476744). The optical densities of bands were analyzed with ImageJ software (RRID: SCR_003070). Since pan-GlyR antibody (mAb4a) detects isoforms of GlyRα subunits, we analyzed the density of assumptive bands indicating 48 kDa GlyRα1 (Becker et al., 1992; Weltzien et al., 2012). The densities of the total GlyR band and the surface GlyR band were normalized to the densities of actin band and total band, respectively.

### Fluorescence recovery after photobleaching and fluorescence loss in photobleaching

For experiments applying fluorescence recovery after photobleaching (FRAP) and fluorescence loss in photobleaching (FLIP; Jaskolski et al., 2009; Hildick et al., 2012), mouse spinal cord neurons (13–15 DIV) were transfected with pCAG-SEP-GlyRα1 (and CMV-mCherry-gephyrin) constructs at 24–48 h prior to experiments. Imaging was performed at room temperature (24 ± 2ºC) in the standard recording solution, and images were acquired with a confocal laser-scanning microscope (Plan Apo-VC 60× H, NA 1.4; model A1, Nikon). Fluorescence of SEP was photobleached using a 488 nm laser at a full power of 40 mW for 62.5 ms, repeated seven times in a 3 × 5 µm rectangular region of interest (ROI) in SEP-expressed neurites. For the focal FRAP protocol, photobleaching was applied to two 1-µm-diameter circular regions of interests with and without mCherry-expressing puncta. Laser power at 1% was used for image acquisitions. In the FRAP-FLIP protocol, repetitive photobleaching at the FLIP regions (3 × 5 µm rectangles) was applied throughout the imaging period directly lateral to a larger initial central bleaching region (10 × 5 µm). The fluorescence intensity of the central ROI (3 × 5 µm) was measured. Since the fluorescence intensity of SEP can be observed in early endosomes, the baseline intensity before photobleaching may contain this fluorescence intensity. Thus, we confirmed surface fractions of SEP fluorescence by extracellular acidification (pH 5.5), which quenches fluorescence after FRAP experiments. In addition, fluorescence in early endosome fractions is photobleached in the ROI; thus, it may not affect fluorescence recovery after photobleaching.

Images were acquired 93 times with 2.9 s intervals in wide FRAP and FRAP-FLIP experiments, and 93 times with 3 s in focal FRAP experiments. The intensity of fluorescence recovery was measured after background subtraction and a correction based on the intensity of the unbleached regions, and was normalized to prebleach intensity. The average fluorescence intensities after photobleaching were fitted by single exponential recovery curves using KaleidaGraph 4.1 (HULINKS). The mobile fraction (MF) was calculated according to Equation 1:

(1) $$MF=(F_{\max}-F_{\left(0\right)})/(F_{pre}-F_{\left(0\right)})$$

where *F*_pre_ is the prebleach intensity, *F*_max_ is the plateau intensity after photobleaching, and *F*_(0)_ is the postbleach intensity at time zero.

For the experiment monitoring fluorescence intensity under local application of glycine, the standard extracellular solution containing both 1 m glycine and Alexa Fluor 594 hydrazide (ThermoFisher Scientific) is applied to single dendrites by glass pipettes (approximate tip diameter, 1 µm) at 2 Hz using a valve-controlled pressure application system (IM300 Microinjector). The area of local glycine application was confirmed by monitoring the fluorescence of Alexa Fluor 564 hydrazide. The fluorescence intensity of SEP-GlyRα1 was measured at gephyrin-positive (GPHN^+^) and gephyrin-negative (GPHN^−^) dendritic shafts, which were covered by glycine application.

### Quantum dot live cell staining, image acquisition, and quantitative analysis

After a single wash with Neurobasal medium, neurons were incubated for 5 min with a primary antibody that recognizes an extracellular epitope of the GlyRα1 subunit (5 µg/ml; mAb2b, Synaptic Systems; RRID: AB_887723), then neurons were washed five times with Neurobasal medium. They were subsequently incubated for 5 min with the secondary antibody [5.2 µg/ml; biotin Fab goat anti-mouse IgG (H+L), Jackson ImmunoResearch; RRID: AB_2338586], then washed again five times. The neurons were next incubated with streptavidin-conjugated quantum dots emitted at 605 nm (1 nm; Life Technologies) in QD binding buffer (Bannai et al., 2006) for 1 min before washing eight times with the standard extracellular solution. Finally, neurons were incubated with FM4-64 (2 µm; Life Technologies) in 40 mm KCl standard solution for 30 s to label active presynaptic sites (Gaffield and Betz, 2006), before returning to the standard external solution. QD labeling, washing, and image acquisition procedures were performed at 37ºC.

QD-labeled neurons were imaged in the standard recording solution using an inverted microscope (model IX70, Olympus) equipped with an oil-immersion objective (100×, NA 1.4), and an EM-CCD camera (ImagEM C9100-13, Hamamatsu Photonics), mercury lamp, appropriate bandpass filter sets for QD 605 (excitation wavelength (ex.) 457/50 and emission wavelength (em.) 605/15), FM4-64 (ex. 510/84 and em.736/128), and Venus (ex. 457/50 and em.530/55). Single images of FM4-64 were taken before the QD imaging. QD image acquisition movies were composed of 300 frames, with each frame being of 67 ms duration and separated by 33 ms, giving a total imaging time of 30 s with MetaMorph software version 7.7 (Molecular Devices; RRID: SCR_002368). The movements of QD-GlyRα1s were recorded within 45 min after labeling. Strychnine (1 µm) was presented only in the cSTR group during staining and imaging.

For single-particle tracking, QD-GlyR particles on Venus-expressing neurons were selectively analyzed. The trajectory of each particle was determined by cross-correlating the image with a Gaussian model of the point spread function (Bonneau et al., 2005), using TI workbench software written by T. Inoue (Waseda University, Tokyo, Japan; Bannai et al., 2009). Only single QDs identified by their characteristic blinking were analyzed, and particles that fluoresced in >295 (of 300) frames were excluded. The synaptic regions were defined by processing FM4-64 images with top-hat filtering (Waataja et al., 2008). For each frame, QD-GlyR fluorescence that merged by at least 1 pixel with the binarized image of FM 4-64 was defined as a synaptic location, and if the QD was >2 pixels away from the FM 4-64 image, this was defined as extrasynaptic. For the calculation of diffusion parameters in the synaptic and extrasynaptic regions, the longest subtrajectories (segments) of single QD-GlyRs that consisted of at least 30 frames in each compartment were used to represent the behavior of each particle in that compartment. Synaptic dwell times were quantified by counting the total number of consecutive frames in which a QD was localized to synaptic regions.

To obtain the diffusion coefficients, values of the mean square displacement (MSD) plot versus time were calculated for each trajectory using Equation 2 (Saxton and Jacobson, 1997), as follows:

(2) $$MSD(n\tau )=\frac{1}{N-n}\overset{N-n}{\underset{i=1}{\sum }}[{\left(x((i+n)\tau )-x(i\tau )\right)}^{2}+{\left(y((i+n)\tau )-y(i\tau )\right)}^{2}],$$

where *τ* is the acquisition time, *N* is the total number of frames, and *n* and *i* are positive integers with *n* representing the time increment. Diffusion coefficients (*D*) were calculated by fitting the first three points of the MSD versus time curves with Equation 3 (Kusumi et al., 1993; Bannai et al., 2006), as follows:

(3) MSD(nτ)=4Dnτ+b,

where *b* is a constant reflecting the spot localization accuracy.

The confinement sizes (*L*) were calculated by fitting the MSD-*n*τ plot of the trajectories showing a restricted motion to Equation 4 (Kusumi et al., 1993), as follows:

(4) $$MSD(n\tau )=\frac{L^{2}}{3}\left(1-\exp\left(-\frac{12Dn\tau }{L^{2}}\right)\right)+4D_{mac}n\tau ,$$

where *L^2^* is the confinement area where diffusion is restricted, and *D*_mac_ is the diffusion coefficient on a long time scale.

### Statistical analysis

Data are presented as the median ± interquartile range for diffusion coefficients obtained from SPT. All other data are expressed as the mean ± SEM. Statistical significance was determined using Student’s unpaired two-tailed *t* test for comparing the reversal potential of glycine-induced currents, for comparing the decay time constant of mIPSCs and for comparing changes in SEP-GlyRα1 fluorescence intensities at gephyrin-positive and gephyrin-negative areas by local glycine application; Student’s paired two-tailed *t* test for comparing fractions of fluorescence recovery between mCherry-GPHN and SEP-GlyRα1; one-way ANOVA with Bonferroni’s *post hoc* test for multiple comparisons for immunostaining experiments and for dwell time and confinement size in SPT experiments; and Kruskal–Wallis test followed by *post hoc* comparisons using Mann–Whitney *U* test with Bonferroni’s correction for other statistical comparisons. Pearson’s correlation coefficient was calculated using linear regression fit on the fractions of fluorescence recovery between mCherry-GPHN and SEP-GlyRα1.

## Results

### Activation-dependent increase in the amplitude of postsynaptic glycine responses

**Figure 1.**
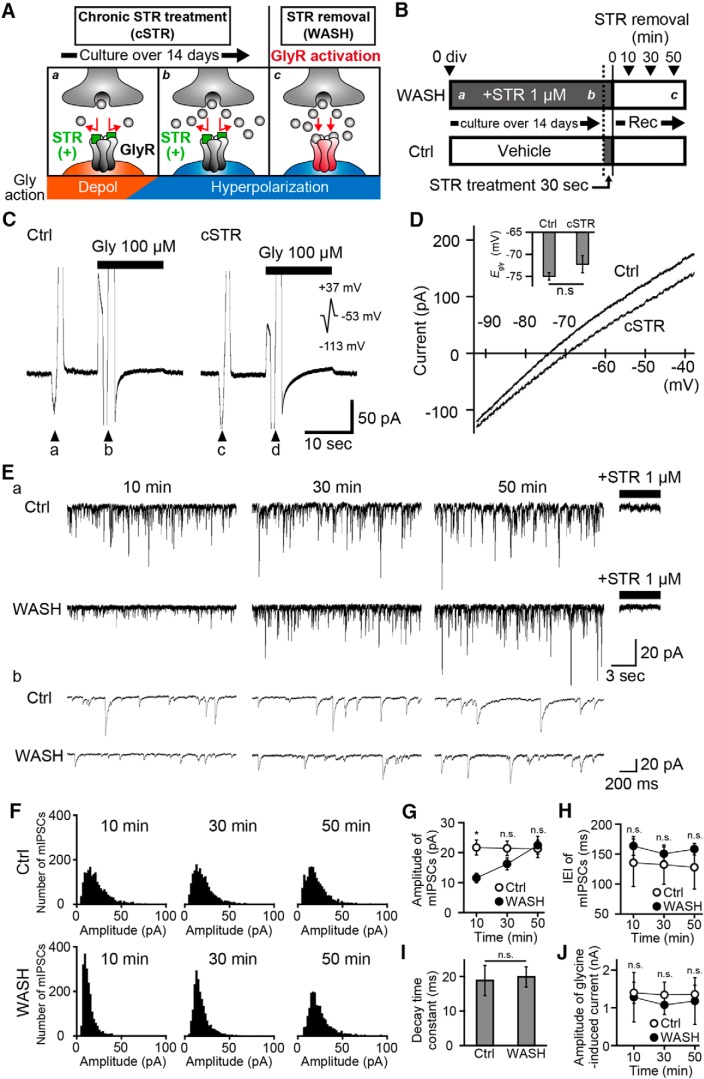
Activation-dependent increases in the amplitude of postsynaptic glycine responses. ***A***, Schematics of the culture model which allows postsynaptic GlyR activation specifically after the D–H shift of the glycinergic response in mature neurons. Gly, glycine; Depol, depolarization. ***B***, Schematic timelines of the experimental protocol for patch-clamp recordings from Ctrl and strychnine-treated neurons (1 μm). Culture medium chronically containing STR from the 0 DIV was exchanged with a recording solution lacking strychnine (WASH). A Ctrl group acutely applied STR for 30 s, and the solution was exchanged with a recording solution lacking strychnine. Patch-clamp recordings were started within 10 min following the washout of strychnine and continued for >50 min after strychnine removal. ***Aa*–*c*** indicate the putative developmental stages shown in ***A***. ***C***, Glycine-induced currents recorded by the gramicidin-perforated patch clamp in Ctrl (left) and in cSTR (right) neurons at a holding potential of −53 mV. Voltage ramp pulses from −113 to +37 mV were applied before (arrowheads, ***a*** and ***c***) and during (arrowheads ***b*** and ***d***) 100 μm glycine application. ***D***, Average *I–V* relationship for glycinergic responses in Ctrl group (*n* = 5 cells) and cSTR group (*n* = 6 cells), as used to derive *E*_gly_. Superimposed histogram shows the mean values of *E*_gly_ ± SEM. ***E***, Representative traces of spontaneous glycinergic mIPSC at three different time points in the Ctrl group (top) and WASH group (bottom). After each recording, strychnine was applied to confirm the strychnine-sensitive glycinergic responses. Representative traces in ***a*** and ***b*** show longer ranges and wide-stretched views from same neurons. ***F***, The amplitude distribution histograms of glycinergic mIPSCs at three different time points (bin width, 2 pA). Each graph is constructed from pooled data of 5 min duration in the Ctrl condition (*n* = 8 cells) and the WASH condition (*n* = 7 cells). ***G***, ***H***, Time courses of mean amplitudes (***G***) and interevent intervals (***H***) of the glycinergic mIPSC in the Ctrl condition (open circles, *n* = 8 cells) and the WASH condition (filled circles, *n* = 7 cells). Each point and bar represents the mean and ±SEM, respectively. ***I***, Mean values ± SEM of the decay time constant of the glycinergic mIPSC in the Ctrl group (*n* = 8 cells) and the WASH group (*n* = 7 cells) at 10 min after strychnine removal. ***J***, Time courses of mean amplitudes of the bath-applied 100 µm glycine-induced IPSC in the Ctrl condition (open circle, *n* = 7 cells) and the WASH condition (filled circles, *n* = 6 cells). Glycine is rapidly applied and removed in the whole-cell body by Y-tube. Each point and bar represents the mean and ±SEM, respectively. The significance of the difference was tested by the Student’s unpaired two-tailed *t* test (***D***, ***I***) and by the Kruskal–Wallis test followed by *post hoc* comparisons using the Mann–Whitney *U* test with Bonferroni’s correction between Ctrl and WASH conditions at each time point (***G***, ***H***, and ***J***). *p < 0.05 (D and I) or 0.05/3 after Bonferroni’s correction (***G–I***). n.s., Not significant.

The present study used a culture model in which initial activation of GlyRs could be pharmacologically delayed until after the developmental shift from depolarizing to hyperpolarizing responses (Fig. 1*A*). Washout of strychnine, which had been applied from the first DIV until each experiment, allowed initial activation of GlyRs once neurons had matured. To validate this model, we first examined whether the cSTR application (1 μm) affected the typical maturation of intracellular Cl^−^ concentrations. We cultured primary spinal cord neurons over 14 DIV and measured the reversal potential of *E*_gly_ using gramicidin-perforated patch-clamp recording (Fig. 1*C*). By subtracting the amplitude of currents that were induced by ramp pulses between the presence and absence of 100 µm glycine (Fig. 1*C*), the *I–V* curves were obtained (Fig. 1*D*). The reversal potentials in cSTR conditions (−70.6 ± 1.9 mV, *n* = 5) were comparable to those in the nonstrychnine Ctrl conditions (−74.2 ± 0.8 mV; *n* = 6; *p* = 0.25; Fig. 1*D*). These results indicate that chronic treatment with strychnine does not affect the development of the intracellular Cl^−^ regulatory system, and that glycinergic transmission after a 2 week culture induces hyperpolarization.

To examine whether the initial activation of postsynaptic GlyRs in the mature stage subsequently modulates glycinergic transmission, we chronically incubated cultures with 1 μm strychnine, from the first DIV until immediately before the experiment (>14 DIV; Fig. 1*B*). Spontaneous mIPSCs were measured by the conventional whole-cell patch-clamp recording in the presence of TTX, CNQX, dl-APV, and SR95531 in the standard bath solution to isolate GlyR responses. In the WASH group, neurons cultured in the chronic presence of strychnine, glycinergic mIPSCs were recorded >60 min after strychnine removal. In the Ctrl group, cultured without strychnine, neurons were transiently incubated with strychnine for 30 s to examine the effect of residual strychnine, before being transferred to the standard bath solution (Fig. 1*B*). The decay time constants of the mIPSCs at 10 min in the WASH group (mean, 19.9 ± 2.9 ms; *n* = 7 cells) were comparable to those in the Ctrl group (mean, 18.8 ± 4.4 ms; *n* = 8 cells; *p* = 0.92, Student’s unpaired two-tailed *t* test; Fig. 1*I*), suggesting that the developmental shift of GlyR subunit compositions was unaffected by chronic strychnine treatment. In the WASH group, the amplitude of glycinergic mIPSCs gradually increased after strychnine removal (11.5 ± 1.6, 16.2 ± 2.0, and 22.4 ± 2.9 pA, respectively, at 10, 30, and 50 min after strychnine removal; *n* = 7; Fig. 1*E–G*). In contrast, the amplitudes of mIPSCs were stable in the Ctrl group across the same recording period (21.7 ± 2.4, 21.4 ± 2.5, and 21.2 ± 2.9 pA, respectively, at 10, 30, and 50 min; *n* = 8; Fig. 1*E–G*). The amplitudes of mIPSCs in WASH groups was significantly lower than those in the Ctrl groups at 10 min (*p* = 0.0037) but were time-dependently increased in the Ctrl group (*p* = 0.094 at 30 min; *p* = 0.779 at 50 min). Hence, glycinergic mIPSCs gradually increase in amplitude following relief from chronic strychnine block, and this is not due to the slow washout of residual strychnine. The interevent intervals (IEIs) of mIPSCs were constant in both the Ctrl conditions (136 ± 16, 132 ± 11, and 128 ± 10 ms, respectively, at 10, 30, and 50 min; *n* = 8) and the WASH conditions (164 ± 39, 150 ± 33, and 158 ± 36 ms, respectively, at 10, 30, and 50 min; *n* = 7). This result may indicate that chronic strychnine treatment has little effect on presynaptic glycine release (Fig. 1*E*,*H*). However, a detailed analysis of synaptic numbers is required since the balance between presynaptic release probability and the number of synapses affects the IEI.

To distinguish whether the gradual increases in the amplitudes of mIPSCs in the WASH group is due to the increase in the number of GlyRs on the whole cell surface or specifically at synapses, 100 µm glycine was locally applied using Y-tube at different time points after strychnine removal. However, the amplitudes of glycine-evoked currents were similar at different time points between the Ctrl group (1.4 ± 0.3, 1.3 ± 0.3, and 1.4 ± 0.2 nA, respectively, at 10, 30, and 50 min after strychnine removal; *n* = 7) and the WASH group (1.3 ± 0.7, 1.1 ± 0.3, and 1.2 ± 0.6 nA, respectively, at 10, 30, and 50 min after strychnine removal; *n* = 8) groups, and no differences were detected between these groups (*p* = 0.23, 0.23, and 0.19, respectively, at 10, 30, and 50 min; Fig. 1*J*), indicating that cell surface expressions of GlyRs did not change during the recording period.

### Chronic blockade of glycine receptors decreases GlyR cluster size at inhibitory synapses

**Figure 2.**
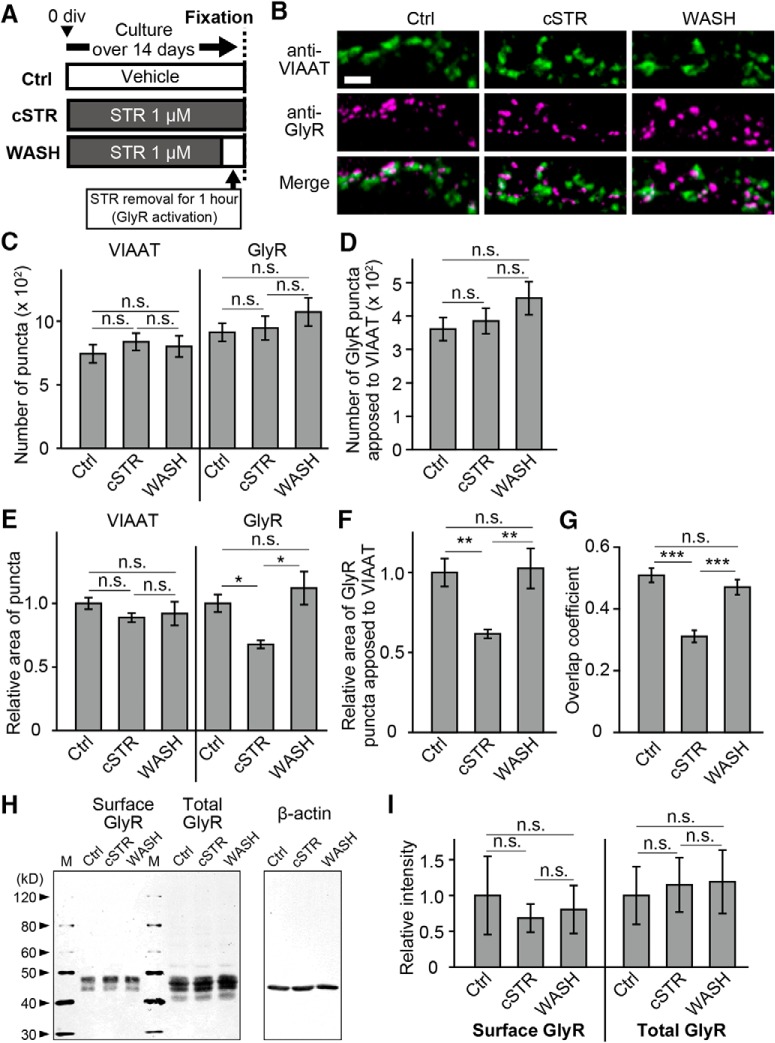
Strychnine removal restores synaptic GlyR clusters. ***A***, Schematic timelines of the experimental protocol. Three experimental groups consisted of neurons incubated with only the vehicle (Ctrl group), 1 μm strychnine chronically (cSTR group), or 1 μm strychnine until 1 h before the experiment (WASH group). Neurons in all groups were subsequently fixed by 4% PFA. ***B***, Representative double-immunostaining images of VIAAT (green) and GlyRs (magenta) in fixed cultured neurons in Ctrl, cSTR, and WASH conditions. Scale bar, 2 μm. ***C***, The number of total VIAAT and GlyR punctate signals per neurons in Ctrl, cSTR, and WASH conditions. ***D***, The number of GlyR punctate signals apposed to VIAAT per neurons in Ctrl, cSTR, and WASH conditions. ***E***, The relative areas of total VIAAT and GlyR punctate signals in Ctrl, cSTR, and WASH conditions. ***F***, The relative areas of GlyR signals apposed to VIAAT in Ctrl, cSTR, and WASH conditions. ***G***, Overlap coefficients of Manders et al. (1993) for VIAAT and GlyR signals. ***H***, A representative immunoblot analysis of total and surface expression levels of glycine receptors in Ctrl, cSTR, and WASH conditions. Biotinylated glycine receptors (surface) were isolated from the detergent-soluble fraction (total). Expression levels of β-actin were used as an internal control for total GlyR. M, Molecular weight marker. ***I***, Quantification of total and surface expression levels of glycine receptors. Expression levels of total GlyRs are normalized by β-actin, and those of surface GlyRs are normalized by total GlyRs. ***C–G***, ***I***, Statistical data are reported as the mean ± SEM derived from 14–17 cells in three independent experiments (***C–G***) and from three independent experiments (***I***). The relative numbers, areas, and intensities are normalized to the mean values of Ctrl conditions. ***C–G***, ***I***, The significance of the difference was tested by one-way ANOVA with Bonferroni’s *post hoc* test (***C–G***) and by Kruskal–Wallis test followed by *post hoc* comparisons using Mann–Whitney *U* test with Bonferroni’s correction (***I***). *p < 0.05, **p < 0.01, ***p < 0.001. n.s. Not significant.

The time-dependent increase in mIPSC amplitudes following receptor blockade may be associated with changes in postsynaptic receptor clusters. To examine this, we prepared three groups of cultured spinal cord neurons: cultures without strychnine (Ctrl), with chronic strychnine (cSTR), and with chronic strychnine for all but the 1 h prior to experiments (WASH; Fig. 2*A*). Localization of glycine receptors at inhibitory synapses was evaluated by double-immunostaining using antibodies against a presynaptic inhibitory terminal marker, the VIAAT (or VGAT), and against the GlyRα1 subunit (mAb2b), a postsynaptic glycine receptor marker (Fig. 2*B*). In the total numbers of VIAAT puncta (cSTR group, 833 ± 67, *n* = 17, *p* = 1.00 vs Ctrl group; WASH group, 798 ± 83, *p* = 1.00 vs Ctrl group; Ctrl group, 739 ± 71) and GlyR puncta (cSTR group, 942 ± 94, *p* = 1.00, vs Ctrl group; WASH group, 1068 ± 110, *p* = 0.76 vs Ctrl group; Ctrl group, 909 ± 72), no differences were detected among the three groups (Fig. 2*C*). Similarly, the numbers of GlyRα1 puncta colocalized to VIAAT puncta were not different among all three conditions (cSTR, 383 ± 38, *p* = 1.00 vs Ctrl; WASH, 451 ± 49, *p* = 0.40, vs Ctrl; Ctrl, 359 ± 34; Fig. 2*D*). Although no differences were observed in the relative size of VIAAT puncta across the three groups (cSTR group, 0.89 ± 0.04, *p* = 0.58 vs Ctrl group; WASH group, 0.92 ± 0.09, *p* = 1.00 vs Ctrl; Ctrl group, 1.00 ± 0.05), the relative size of GlyR puncta was significantly smaller in the cSTR group than those in Ctrl and WASH groups (cSTR group, 0.68 ± 0.03, *p* = 0.02 vs Ctrl; WASH group, 1.12 ± 0.13, *p* = 0.98 vs Ctrl; Ctrl group, 1.00 ± 0.07; Fig. 2*E*). In addition, the relative size of GlyRα1 puncta apposed to VIAAT signals in the cSTR group (0.62 ± 0.03) was significantly smaller than those of GlyRα1 puncta in both the Ctrl group (1.00 ± 0.09; *p* = 0.007) and the WASH group (1.03 ± 0.13; *p* = 0.004; Fig. 2*F*). In addition, the extent of colocalization of the VIAAT and GlyRα1 puncta was significantly lower in the cSTR group (overlap coefficient, 0.31 ± 0.02) compared with that in the Ctrl group (0.51 ± 0.02; *p* < 0.0001) and the WASH group (0.47 ± 0.02; *p* < 0.0001; Fig. 2*G*). The total number of GlyRα1 proteins expressed in the cells (cSTR group, *p* = 1.00 vs Ctrl group; WASH group, *p* = 1.00 vs Ctrl group), or the total number of GlyRα1 proteins expressed on the plasma membrane were not significantly different across all three experimental groups, as measured by Western blotting (cSTR group, *p* = 1.00 vs Ctrl group; WASH group, *p* = 1.00 vs Ctrl group; Fig. 2*H*,*I*). This indicates that chronic treatment with strychnine reduces the size of GlyR clusters localized at inhibitory synapses, without affecting the numbers of total and synaptic GlyR clusters. The distribution of GlyR cluster size and localization returns to control levels within 1 h of strychnine washout. Hence, in these mature cultures, synaptic GlyR cluster size is rapidly increased following receptor activation, coincident with a change in surface GlyR localization.

In addition, chronic strychnine treatment does not affect the synaptic number (Fig. 2*D*). Thus, the comparable length of IEIs in mIPSCs between the WASH and Ctrl groups (Fig. 1*E*,*H*) indicates that presynaptic release probability is unaffected by chronic strychnine treatment.

### GlyR activation reduces the spatial dynamics of laterally diffusive GlyRs in the cytoplasmic membrane

**Figure 3.**
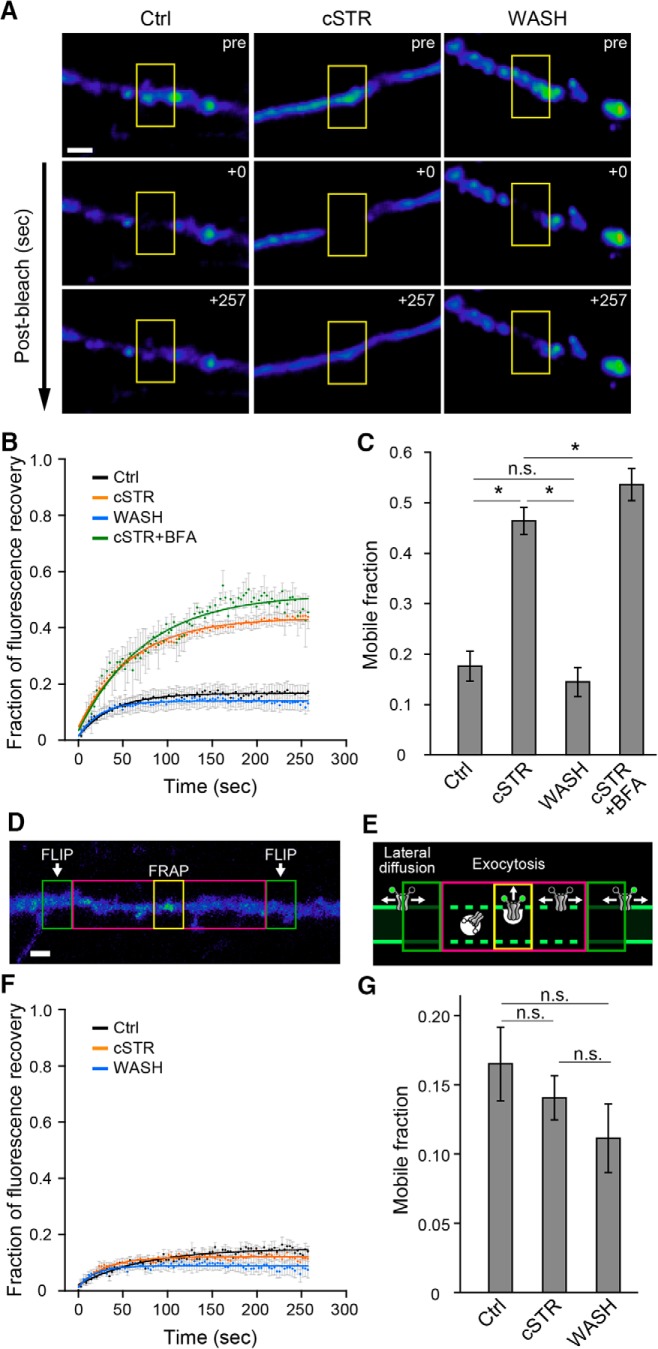
Cell surface dynamics of glycine receptors in live neurons. ***A***, FRAP in neuronal processes expressing SEP-GlyRα1. Fluorescence of SEP-GlyRα1 was bleached in the region of the neuronal process indicated by the yellow rectangles. Each row represents before (pre), immediately after (+0 s), and at a later time (+257 s) after photobleaching in each condition (Ctrl, cSTR, and WASH). Scale bar, 2 µm. ***B***, Normalized fluorescence recovery curves after photobleaching in Ctrl (black, *n* = 15 cells), cSTR (orange, *n* = 16 cells), WASH (cyan, *n* = 12 cells), and cSTR+ BFA (green, *n* = 5 cells) conditions. Each plot and bar represents the mean and ±SEM. ***C***, Averaged mobile fractions in Ctrl, cSTR, WASH, and cSTR with BFA conditions (mean ± SEM), as shown in ***B***. The mobile fraction was defined as the extent of fluorescence recovery at the end of the imaging time. ***D***, A representative image of SEP-GlyRα1 fluorescence and the regions of the neuronal processes used for the FRAP-FLIP experiments. Repetitive photobleaching (FLIP, green rectangles) occurred at regions bilateral to the central FRAP region (yellow rectangle). A magenta rectangle shows the buffer region used to minimize any effects of the leakage of light from the FLIP regions. Scale bar, 2 µm. ***E***, A schematic of hypothetical SEP-GlyRα1 movements under the FRAP-FLIP configuration shown in ***D***. ***F***, Normalized fluorescence recovery curves (mean ± SEM) in Ctrl (*n* = 8 cells, black), cSTR (*n* = 15 cells, orange), and WASH (*n* = 12 cells, cyan) conditions in the FRAP-FLIP experiments. ***G***, Averaged mobile fractions in the Ctrl, cSTR, and WASH conditions (mean ± SEM), as shown in ***F***. The significance of difference was tested by Kruskal–Wallis test followed by *post hoc* comparisons using the Mann–Whitney *U* test with Bonferroni’s correction. ***p < 0.001/6 after Bonferroni’s correction for the six tests. n.s., Not significant.

The increase in GlyR cluster size and change in surface localization without any apparent change in total cellular or surface expression of GlyRs suggests that GlyR activation somehow changes GlyR surface distribution to recruit receptors to synaptic clusters. To examine the behavior of surface-localized GlyRs, we applied FRAP to single neuronal processes (Soumpasis, 1983; Reits and Neefjes, 2001). To selectively visualize surface GlyRs, we generated a GlyRα1 construct tagged with SEP to give a pH-sensitive enhanced green fluorescent protein sequence in the extracellular N-terminal domain of GlyRα1 subunit (SEP-GlyR). SEP emits a distinctively high fluorescence when in a neutral pH environment but does not fluoresce when within a more acidic environment, such as that found inside intracellular transport organelles, and hence this enables the selective visualization of the surface population of proteins (Miesenböck et al., 1998; Sankaranarayanan and Ryan, 2001; Ashby et al., 2004). The fluorescence intensity of SEP-GlyRs on single neuronal processes in the three groups was measured before and after photobleaching (Fig. 3*A*). Localized photobleaching eliminates fluorescent signals from SEP-GlyRs within the targeted region, and hence recovery of the fluorescent signal reflects the movement of unbleached SEP-GlyRs into the target region, a so-called “mobile fraction,” that was quantified as the relative recovery of fluorescence at the end of the 257 s imaging period after photobleaching (Fig. 3*B*,*C*). In neurons from the Ctrl group, the mobile fraction reached a plateau of 0.18 ± 0.03 (*n* = 15), which was similar to that observed in the WASH group (0.14 ± 0.03; *n* = 12; *p* = 0.52). In contrast, the mobile fraction in the cSTR group was significantly higher (0.46 ± 0.03; *n* = 16; *p* < 0.0001), indicating an increase in the population of new or mobile SEP-GlyRs that reappear at the neurite plasma membrane after photobleaching. Conversely, the lower mobile fractions in the Ctrl and WASH groups suggest that GlyRs in these groups are less mobile or that receptor activation makes them more stable.

The increase in GlyR-SEP fluorescence after photobleaching may arise from either (1) exocytosis of GlyRs from cytoplasmic vesicles into the plasma membrane or (2) lateral diffusion of surface GlyRs from outside of the bleached region. To distinguish which of these is increased by chronic strychnine administration, we measured the fluorescence recovery in chronically strychnine-treated neurons in the presence of BFA, an inhibitor of ER–Golgi transport that blocks exocytosis (Fujiwara et al., 1988; Rannals and Kapur, 2011). BFA (5 µg/ml) was added to the external solution for 90 min before each imaging. The mobile fraction in the presence of BFA was also elevated relative to control and was not significantly different from that seen in the cSTR group (cSTR plus BFA: 0.54 ± 0.03, *n* = 5, *p* = 0.11; Fig. 3*B*,*C*).

We therefore next examined whether the higher mobile fraction of SEP-GlyR in the cSTR group is due to an increase in the appearance of new GlyRs from lateral diffusion, using a combination of FRAP with FLIP (Jaskolski et al., 2009; Hildick et al., 2012). FLIP involved repeated photobleaching of regions bilateral to the center region targeted by FRAP, so that GlyRs moving into the center target region are photobleached before they arrive (Fig. 3*D*,*E*). Thus, FLIP excludes laterally diffusing GlyRs from the mobile fraction, leaving just newly exocytosed SEP-GlyRα1 to contribute to the fluorescence recovery. The mobile fractions in these FRAP-FLIP experiments were comparable among the experimental groups (cSTR group, 0.14 ± 0.02, *n* = 13, *p* = 0.70 vs Ctrl group; WASH group, 0.11 ± 0.02, *n* = 11, *p* = 0.09 vs Ctrl group; Ctrl group, 0.17 ± 0.03, *n* = 8; Fig. 3*F*,*G*). Together, these results demonstrate that chronic strychnine administration does not affect the rate of exocytosis of new GlyRs from intracellular stores into the plasma membrane but, rather, increases the lateral diffusion of GlyRs along the plasma membrane.

### GlyR dynamics at gephyrin-expressing postsynapses

**Figure 4.**
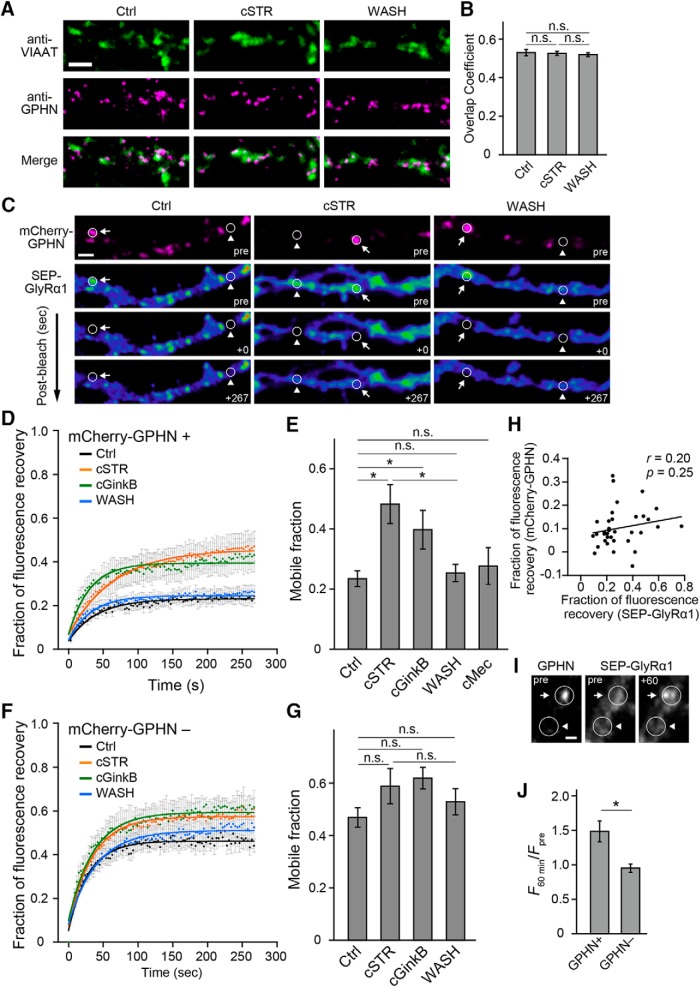
Glycine receptor dynamics at gephyrin-identified synaptic zones. ***A***, Representative double-immunostaining images of VIAAT (green) and GPHN (red) in fixed cultured neurons in Ctrl, cSTR, and WASH conditions. Scale bar, 2 μm. ***B***, Overlap coefficients of Manders et al. (1993) of VIAAT and GPHN signals (*n* = 13–16 cells). ***C***, Representative images of neuronal processes coexpressing SEP-GlyRα1 and mCherry-GPHN in Ctrl, cSTR, and WASH conditions. Top, Expression of mCherry-GPHN fluorescence. FRAP of SEP-GlyRα1 was measured at focal spots with (arrows) and without (arrow heads) mCherry-GPHN signals. Each row represents before (pre), immediately after (+0 s), and at a later time (+267 s) after photobleaching. Scale bar, 2 µm. ***D***, Fluorescence recovery curves after photobleaching at focal spots expressing mCherry-GPHN in control (black), cSTR (orange), cGinkB (green), and WASH (cyan) conditions. Each point and bar represents the mean and ±SEM, respectively. ***E***, Averaged mobile fractions at focal spots expressing mCherry-GPHN in Ctrl (*n* = 14 cells), cSTR (*n* = 8 cells), cGinkB (*n* = 8 cells), WASH (*n* = 15 cells), and cMEC (*n* = 8 cells) conditions (mean ± SEM) for the data shown in ***D***. ***F***, Fluorescence recovery curves after photobleaching at mCherry-GPHN^−^ regions in control (black), cSTR (orange), cGinkB (green), and WASH (cyan) conditions. Each point and bar represents mean and ±SEM, respectively. ***G***, Average mobile fraction at focal spots lacking mCherry-GPHN in Ctrl (*n* = 14 cells), cSTR (*n* = 8 cells), cGinkB (*n* = 8 cells), and WASH (*n* = 15 cells) conditions (mean ± SEM), for the data shown in ***F***. ***H***, The correlation between fractions of fluorescence recovery of SEP-GlyRα1 and mCherry-GPHN (*n* = 37 cells). Each plot represents individual samples in Ctrl, cSTR, and WASH groups. ***I***, Representative fluorescence images of mCherry-GPHN and SEP-GlyRα1 at a single dendrite before (pre) and after (+60) local application of glycine for 60 min. Arrows and arrowheads shows GPHN^+^ and GPHN^−^ zones. Scale bar, 1 µm. ***J***, Average changes of SEP-GlyRα1 fluorescence intensity in the GPHN^+^ and GPHN^−^ zones at single dendrite (*n* = 7 cells). The significance of the difference was tested by one-way ANOVA with Bonferroni’s *post hoc* test (***B***), by Kruskal–Wallis test followed by *post hoc* comparisons using the Mann–Whitney *U* test with Bonferroni’s correction (***E***, ***G***) and Student’s paired (***H***) or unpaired (***J***) two-tailed *t* test. The Pearson’s correlation coefficients (*r*) and *p* values (*p*) are indicated in ***H***. *p < 0.05/10 after Bonferroni’s correction for the 10 tests (***E***) and *p* < 0.05 (***J***). n.s., Not significant.

Changes of the diffusion properties of receptors can determine the receptor localization at synapses (Dahan et al., 2003; Bannai et al., 2009; Choquet and Triller, 2013). The postsynaptic localization and cluster sizes of GlyRs are regulated by gephyrin, a major scaffolding protein of GlyRs (Kirsch and Betz, 1995; Calamai et al., 2009). The distribution of gephyrin has been reported to be either affected (Kirsch and Betz, 1998) or unaffected (Lévi et al., 1998; Tyagarajan and Fritschy, 2014) by GlyR activation. Hence, we examined whether chronic strychnine treatment affects the distribution of gephyrin at inhibitory synapses in the present study by measuring the overlap coefficient of Manders et al. (1993) between immunostained gephyrin and VIAAT puncta. Neither chronic strychnine application (cSTR group) nor the wash from chronic strychnine with subsequent GlyR activation (WASH group) affected the gephyrin distribution (cSTR group, 0.53 ± 0.01, *p* = 1, *n* = 15 vs Ctrl group; WASH group, 0.52 ± 0.01, *p* = 0.57, *n* = 16, vs Ctrl group; Ctrl group, 0.54 ± 0.01, *n* = 13; Fig. 4*A*,*B*).　

Next, we examined whether the GlyR activation-induced change in GlyR mobility was selective for regions around inhibitory postsynapses, as defined by gephyrin-positive locations. Neuronal cultures were cotransfected with both SEP-GlyRα1 and mCherry-GPHN, and the fluorescence intensity of SEP-GlyRα1 was measured before and after photobleaching and was compared between mCherry-GPHN-positive and mCherry-GPHN-negative regions (Fig. 4*C*). At mCherry-GPHN-positive spots, the mobile fraction of SEP-GlyRα1 was larger in the chronic presence of strychnine (cSTR; 0.48 ± 0.06, *n* = 8, *p* = 0.002) in comparison with the mobile fraction of SEP-GlyRα1s at mCherry-GPHN-positive spots in Ctrl cultures (0.23 ± 0.03, *n* = 14). Although ectopic expression of SEP-GlyRα1s are likely to form homomeric GlyRs, synaptic localization of GlyRs is regulated by interactions between gephyrin and heteromeric GlyRs containing α1- and β-subunits (Tyagarajan and Fritschy, 2014). Thus, we also measured the mobile fraction of SEP-GlyRα1 in the chronic presence of ginkgolide B (cGinkB), a potent channel blocker of heteromeric GlyR (Kondratskaya et al., 2005; 0.40 ± 0.06, *n* = 8) and confirmed that it was significantly larger than that in the Ctrl group (*p* = 0.005). In contrast, the mobile fraction in the WASH group (0.25 ± 0.03, *n* = 15) was similar to that in the Ctrl group (*p* = 0.62; Fig. 4*D*,*E*). However, the mobile fractions at mCherry-GPHN-lacking regions were all relatively high, with no significant differences among the groups (Ctrl group, 0.47 ± 0.04, *n* = 14; cSTR group, 0.59 ± 0.07, *n* = 8; cGinkB group, 0.62 ± 0.04, *n* = 8; WASH group, 0.53 ± 0.05, *n* = 15; Fig. 4*F*,*G*). Although high-power excitation laser light also decreased the fluorescence intensity of mCherry-GPHN, the mobile fractions of fluorescence recovery between SEP-GlyRα1 and mCherry-GPHN in the same FRAP regions in the sum of the three experimental groups were not correlated (*r* = 0.20, *p* = 0.25, *n* = 37; Fig. 4*H*). Thus, mobile fractions of SEP-GlyRα1 were independent of the photobleaching of mCherry-GPHN. These results suggest that the lateral mobility of GlyRs on the cell surface under intact glycinergic transmission is lower at sites of gephyrin localization, and this receptor stabilization is absent in chronically inactivated GlyRs. In addition, 1 µm strychnine is reported to affect nicotinic acetylcholine receptors (nAChRs; Matsubayashi et al., 1998). However, the mobile fraction of SEP-GlyRα1 in the chronic presence 0.5 µm mecamylamine hydrochloride (cMec), a noncompetitive antagonist of nAChRs (Papke et al., 2001; Yasuyoshi et al., 2002; cMec, 0.28 ± 0.06, *n* = 8) was comparable to that in the Ctrl group (*p* = 0.62) in mCherry-GPHN-positive regions (Fig. 4*E*). Thus, higher GlyR mobility in the cSTR group is independent of nAChR blockade.

We next confirmed whether the local glycine application directly and selectively induces GlyR clustering at the gephyrin-positive area. The 1 m glycine was applied using a micropipette placed beside single dendrites in cSTR group. After glycine application for 60 min, the fluorescence intensity of SEP-GlyRα1 was increased at gephyrin-positive spots, and the changes are significantly larger in the GPHN^+^ area (1.49 ± 0.15, *n* = 7 cells) than in the GPHN^−^ area (0.95 ± 0.06, *n* = 7 cells; *p* = 0.01; Fig. 4*I*,*J*). These results suggest that activated GlyRs make clusters selectively at gephyrin-expressing postsynapses within 60 min.

### Glycinergic transmission regulates the formation of synaptic GlyR clusters, but not their maintenance

**Figure 5.**
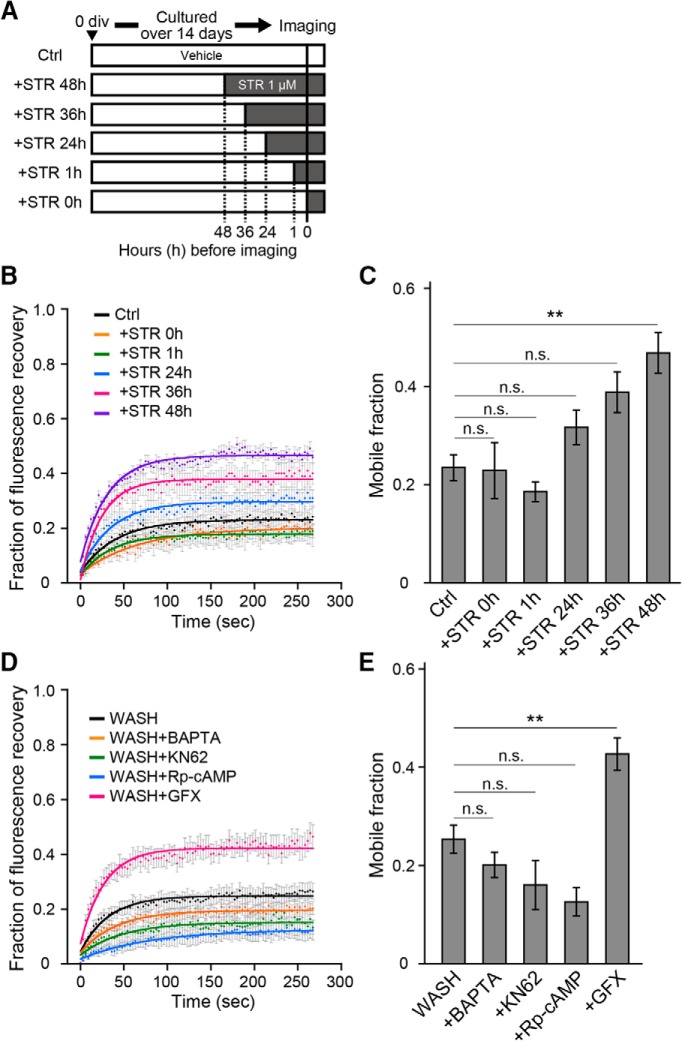
GlyR activation regulates the formation, but not the maintenance, of GlyR clustering. ***A***, Schematic timelines of strychnine treatments on mature neurons. Neurons cultured over 14 DIV without previous application of strychnine were applied for the administration of 1 μm strychnine for 48 h (+STR 48h), 36 h (+STR 36h), 24 h (+STR 24h), 1 h (+STR 1 h), or immediate (+STR 0 h) before and during imaging. ***B***, Normalized fluorescence curves at focal spots with mCherry-GPHN signals in Ctrl (*n* = 14 cells, black), Ctrl with acute (+STR 0h; *n* = 6 cells, orange), with 1 h (+STR 1h; *n* = 9 cells, green), with 24 h (+STR 24h; *n* = 15 cells, cyan), with 36 h (+STR 36h; *n* = 11 cells, magenta), and with 48 h (+STR 48h; *n* = 10 cells, purple) of STR treatment before each experiment. Each point and bar represents mean and ±SEM, respectively. ***C***, Average mobile fractions (mean ± SEM) shown in ***B***. ***D***, Fluorescence recovery curves after photobleaching at focal spots expressing mCherry-GPHN in WASH (*n* = 15 cells, black, shown in Fig. 4*D*), WASH with BAPTA-AM (*n* = 5 cells, orange), WASH with KN62 (*n* = 6 cells, green), WASH with Rp-cAMP (*n* = 5 cells, cyan), and WASH with GFX (*n* = 11 cells, magenta) conditions. Each point and bar represents mean and ±SEM, respectively. ***E***, Averaged mobile fractions at focal spots expressing mCherry-GPHN in WASH (*n* = 15 cells, shown in Fig. 4*E*), WASH with BAPTA-AM (*n* = 5 cells), WASH with KN62 (*n* = 6 cells), WASH with Rp-cAMP (*n* = 5 cells), and WASH with GFX (*n* = 11 cells) conditions (mean ± SEM), for the data shown in ***D***. The significance of difference was tested by the Kruskal–Wallis test followed by *post hoc* comparisons using the Mann–Whitney *U* test with Bonferroni’s correction. **p < 0.01/15 and *p* < 0.01/10 after Bonferroni’s correction for the 15 and 10 tests, respectively. n.s., Not significant.

The results above indicate that GlyR activation is required for the formation of large, stable GlyR clusters at gephyrin-positive synapses, but it is not clear whether stabilized GlyRs remain synapses only during the period they are activated or are stabilized for a longer duration without further activation. Then, after 14 d of neuronal culture in the absence of any GlyR block, we applied 1 µm strychnine for 48, 36, 24, and 1 h or just immediately (0 h) prior to acquiring imaging data (Fig. 5*A*). FRAP was used to measure SEP-GlyRα1s fluorescence intensity at mCherry-GPHN^+^ spots. The results showed no significant differences were found in the mobile fractions between the groups treated 36 h or shorter duration as compared to control (STR0h, 0.23 ± 0.06, *n* = 6, *p* = 0.78; STR1h, 0.19 ± 0.02, *n* = 9, *p* = 0.31; STR24h, 0.32 ± 0.04, *n* = 15, *p* = 0.07, STR36h, 0.39 ± 0.04, *n* = 11, *p* = 0.02, compared to Ctrl, 0.23 ± 0.03, *n* = 14; Fig. 5*B*,*C*). In contrast, the mobile fraction of strychnine treatment for 48 h (0.47 ± 0.04, *n* = 10) significantly increased in comparison to control (*p* = 0.0001). Hence, the block of previously activated GlyRs for up to 24 h has little effect, but for 48 h the block increases GlyR mobility at synapses.

### Ca^2+^ and CaMKII-independent clustering of synaptic GlyRs

Previous reports have demonstrated that synaptic GlyR accumulation in developing neurons requires Ca^2+^ influx and CaMKII activity (Kirsch and Betz, 1998; Lévi et al., 2008; Yamanaka et al., 2013). Thus, we also examined these in mature neurons, directly measuring whether the activation-induced GlyR stabilization was affected by a cell-permeable Ca^2+^ chelator (BAPTA-AM), and a CaMKII inhibitor (KN62), applying these treatments during the 1 h washout of chronic strychnine treatment. The mobile fraction after incubation with either BAPTA-AM (0.20 ± 0.03, *n* = 5, *p* = 0.50) or KN62 (0.16 ± 0.05, *n* = 6, *p* = 0.05) was not significantly different from that in the WASH group (0.25 ± 0.03, *n* = 15; Fig. 5*D*,*E*), indicating that intracellular Ca^2+^ or CaMKII is not a prerequisite for the GlyR activation-induced decrease in lateral mobility. We further tested the involvement of protein kinase A (PKA) or protein kinase C (PKC) on the activation-induced GlyR clustering. The effect of phosphothioate-cAMP (Rp-cAMP), a cell-permeable cAMP analog antagonizing PKA activity, and GFX, a cell-permeable PKC inhibitor, are tested by application during the 1 h GlyR activation after the washout of chronic strychnine. The administration of Rp-cAMP showed no difference (0.13 ± 0.14, *n* = 5, *p* = 0.02), but administration of GFX (0.43 ± 0.03, *n* = 11, *p* = 0.0008) induced a significantly higher mobile fraction compared with that in the WASH group (0.25 ± 0.03, *n* = 15; Fig. 5*D*,*E*).

### Single-particle tracking of endogenous GlyRs at synapses

**Figure 6.**
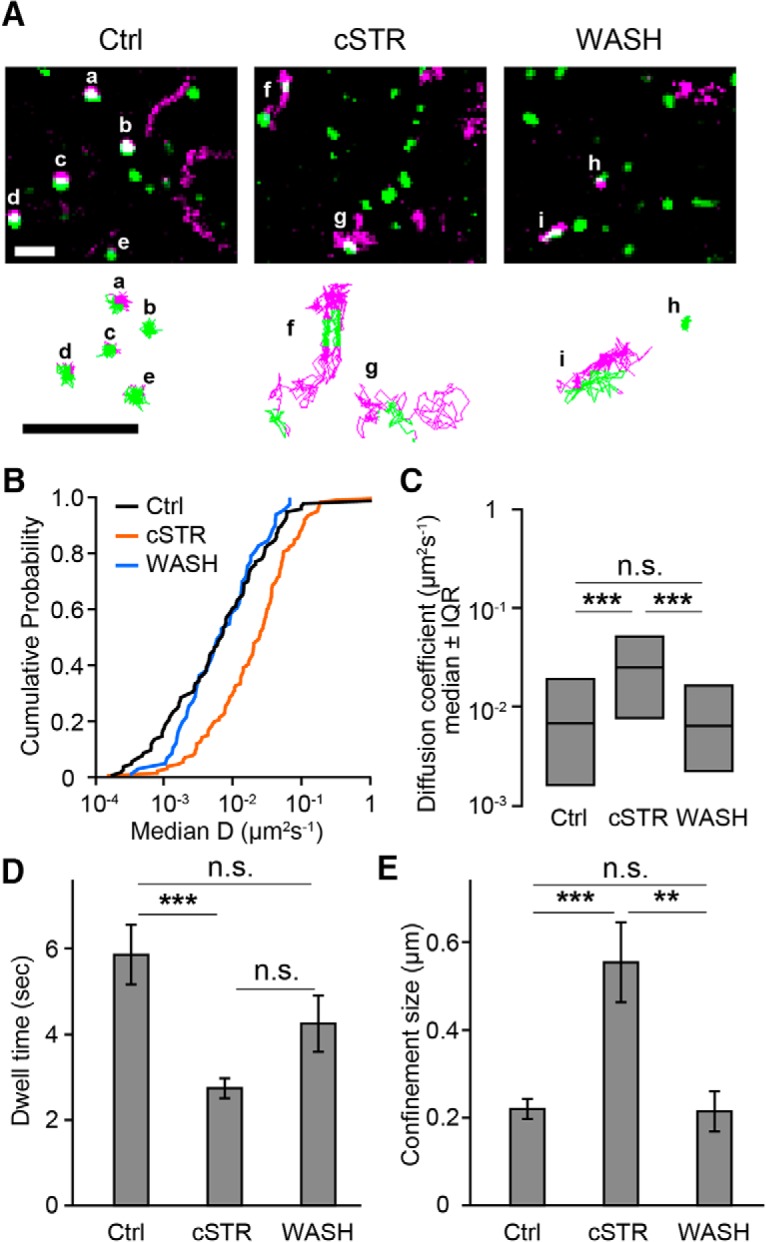
GlyR activation modulates its diffusion properties around active synapses. ***A***, Representative images of QD-labeled endogenous GlyRs (magenta) and FM4-64-labeled active synapses (green) shown as maximum intensity projections of 30 s of image recordings in Ctrl, cSTR, and WASH conditions (top panels). The bottom panels show the trajectories of correspondent QD-labeled GlyRs (centroids) shown in the top panels inside (green) and outside (magenta) active synapses. Scale bars, 2 µm. ***B***, Cumulative probabilities of the median diffusion coefficient of each QD-GlyR within the active synapse region in Ctrl (*n* = 101 particles, 7 cells), cSTR (*n* = 125 particles, 8 cells), and WASH (*n* = 54 particles, 7 cells) conditions. ***C***, Median diffusion coefficients of each QD-GlyR at synapses comparing Ctrl, cSTR, and WASH conditions (median ± 25–75% interquartile range (IQR)). ***D***, Average dwell times of QD-GlyRs within synapse regions (Ctrl: *n* = 150 particles, 7 cells; cSTR: *n* = 314 particles, 8 cells; WASH: *n* = 97 particles, 7 cells, mean ± SEM). ***E***, Average confinement size of QD-GlyRs at synapses (mean ± SEM) in Ctrl (*n* = 49 particles, 7cells), cSTR (*n* = 38 particles, 8 cells), and WASH (*n* = 19 particles, 7 cells) conditions. Significance of difference was tested by Kruskal–Wallis test followed by *post hoc* comparisons using the Mann–Whitney *U* test with Bonferroni’s correction, ***p < 0.001/3 after Bonferroni’s correction for the three tests (***C***) and by one-way ANOVA with Bonferroni *post hoc* test (***D***, ***E***). **p < 0.01, ***p < 0.001. n.s., Not significant.

The results above suggest that the mobility of synaptic GlyRs is dynamically modulated by its activation. To more directly measure the lateral diffusion of GlyRs at synapses, we labeled the α1-GlyR subunit with fluorescent QDs (Dahan et al., 2003; Lévi et al., 2008). Active synaptic regions were identified by FM4-64, a lipophilic styryl dye that gets incorporated into synaptic vesicles via endocytosis (Gaffield and Betz, 2006). Individual QD-GlyRs showed quite variable trajectories and rates of diffusion in each experimental group, although in the Ctrl and WASH groups trajectories were largely restricted to the areas around active synaptic zones (Fig. 6*A*). The median QD-GlyR diffusion coefficients in the Ctrl group (6.95 × 10^−3^ µm^2^s^−1^, *n* = 101 particles) and the WASH group (6.39 × 10^−3^ µm^2^s^−1^, *n* = 54 particles) were similar (*p* = 0.79) but significantly lower than the median diffusion coefficient of GlyRs in the cSTR group (2.46 × 10^−2^ µm^2^s^−1^, *n* = 125 particles; Ctrl vs cSTR, *p* < 0.0001; WASH vs cSTR, *p* < 0.0001; Fig. 6*B*,*C*). These results suggest that inactive GlyRs are highly diffusive compared with activated GlyRs, with diffusion rates returning to control values within 1 h of receptor activation.

To determine the effects of receptor activation on the mobility of QD-GlyRs localized at synapses, we quantified the synaptic dwell time and the confinement size of each QD-GlyR in synaptic domains. Dwell time was calculated as the longest sum of consecutive subtrajectories in which each QD-GlyR stayed within the active synaptic zone (defined as the overlap with FM4-64 fluorescence; see Materials and Methods). The average dwell time in cSTR group (2.7 ± 0.2 s, *n* = 314 particles) was shorter than that of Ctrl group (5.9 ± 0.7 s, *n* = 150 particles; *p* < 0.0001). The average dwell time in the WASH group (4.3 ± 0.7 s, *n* = 97 particles) returned to that seen in the Ctrl group, although it was not significantly different from that in the cSTR group (*p* = 0.09; Fig. 6*D*). The confinement size of each QD-GlyR, defined as the length of an equivalent square within which diffusion was restricted (see Materials and Methods), was significantly larger in the cSTR condition (0.55 ± 0.09 µm, *n* = 38 particles) compared with that in the Ctrl condition (0.22 ± 0.02 µm, *n* = 45 particles; *p* = 0.0002) and the WASH condition (0.21 ± 0.05 µm, *n* = 19 particles; *p* = 0.004; Fig. 6*E*). Hence, chronically blocked (inactive) GlyRs diffuse at a faster rate and over a larger area in synaptic domains compared with activated GlyRs. Inactive GlyRs also reside for briefer durations at synapses.

## Discussion

The present study demonstrated here that glycinergic synaptic responses and spatial distributions of GlyRs were modulated by receptor activation via presynaptically released glycine in mature spinal cord neurons independent of the Ca^2+^-mediated mechanism. The clustering of other ionotropic receptors, such as AMPA receptors and GABA_A_ receptors, are regulated by a Ca^2+^-dependent process in both immature and mature neurons (Borgdorff and Choquet, 2002; Petrini et al., 2014). As for GABA_A_ receptors, for example, excitatory activity induces depolarization and increases clustering of GABA_A_ receptors via CaMKII activity (Petrini et al., 2014). The prevailing concept for GlyR synaptic clusters that was developed from studies on immature circuits is similar to that for AMPA receptors and GABA_A_ receptors. Glycine receptor activation induces depolarization in immature neurons and subsequent Ca^2+^ influx (Kirsch and Betz, 1998), which activates Ca^2+^-dependent kinases to elicit increases in receptor clusters (Charrier et al., 2010; Yamanaka et al., 2013). This concept relies on the fact that glycine and GABA_A_ receptor activation induces depolarizations in early development, as intracellular Cl^−^ is elevated due to the absence of functional K^+^-Cl^−^ cotransporter 2 (KCC2). Since KCC2 is expressed during development, the resultant Cl^−^ efflux lowers intracellular Cl^−^ and converts the GABA and glycine response to hyperpolarizing (Kakazu et al., 1999; Rivera et al., 1999). This upregulation of KCC2 and switch from depolarization to hyperpolarization occurs over the first postnatal weeks in most regions of the central nervous system (Ehrlich et al, 1999; Jean-Xavier et al., 2006). In cultured neurons of spinal cord and brainstem, the switch from GABA or glycine-induced depolarizations to hyperpolarizations occurs between 1 and 3 weeks *in vitro*, with the precise timing depending on culture conditions (Chen et al., 1996; Khirug et al., 2005). In our experiments, the glycine reversal potential after 14 d of culture was already hyperpolarizing and, more importantly, was the same as that in cultures in which GlyRs were chronically blocked with strychnine (Fig. 1*D*). Thus, our culture model system mimicked the first activation of newly expressed GlyRs on the cytoplasmic membrane after maturation of Cl^−^ homeostasis when glycinergic transmission becomes dominant from GABAergic transmission in physiological conditions (Kotak et al., 1998; Gao et al., 2001; Russier et al., 2002). Indeed, the present results demonstrate that the activation-induced decrease of synaptic GlyR mobility that was associated with activation-dependent GlyR aggregation at synapses was not affected by Ca^2+^ chelation and inhibition of CaMKII (Fig. 5*D*,*E*). Although the activity-dependent cluster stabilization at the mature stage that we demonstrated here shares similarities with previous reports during early development, the underlying mechanisms must be distinct due to the lack of depolarization. Accordingly, our results propose that presynaptic release of inhibitory neurotransmitters alone is sufficient to regulate postsynaptic localization of inhibitory neurotransmitter receptors. Furthermore, this Ca^2+^-independent clustering of inhibitory neurotransmitter receptors might contribute to the homeostatic turnover of the postsynaptic receptors in mature synapses.

Neural activity and NMDA receptor activation decreases the lateral diffusion of GlyRs and increases their accumulation into synaptic clusters (Kirsch and Betz, 1998; Lévi et al., 2008). A remarkable observation in the present study is the speed at which this can occur. Only 1 h of GlyR activation was sufficient to restrict GlyR diffusion to synapses and to stabilize and increase functional clusters. Our experiments were all conducted in the continued presence of TTX, dl-APV, CNQX, and SR95531, excluding any more general changes in neural activity and excitatory receptor activation as contributors to this receptor stabilization. In addition, the interevent intervals of mIPSCs were not affected by chronic strychnine administration, indicating no prolonged changes in the neural circuit excitability (Fig. 1*H*). Hence, GlyR activation by itself stabilizes synaptic GlyRs, independent of changes in overall levels of excitatory neural activity.

**Figure 7.**
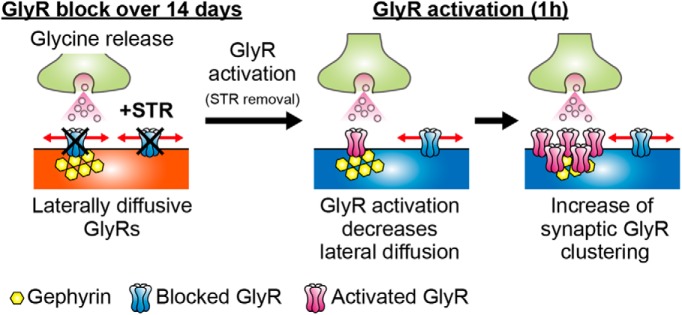
Summary diagram of activation-dependent postsynaptic GlyR clustering. (Left) Antagonized GlyRs by chronic application of strychnine (STR) are highly diffusive even at synaptic site in the presence of gephyrin, a scaffolding protein. (Middle) Strychnine removal allows GlyRs to be activated and decreases lateral diffusion at synaptic sites. (Right) Density of GlyRs increases at synaptic sites due to relatively lower diffusive properties than the extrasynaptic area.

GlyR diffusion properties and synaptic localization is known to depend critically on interactions with gephyrin, the scaffolding protein of inhibitory neurotransmitter receptors (Tyagarajan and Fritschy, 2014). In the present study, direct measurement of endogenous GlyR movements by single-particle tracking demonstrated that chronic receptor inactivation resulted in larger diffusion coefficients and an increase in the area over which GlyRs moved (Fig. 6*B*,*C*,*E*). When GlyR activation was allowed, the diffusive property of those GlyRs at synaptic domains decreased (Figs. 6, 7). The restricted mobility of activated GlyRs at synapses is likely to also involve interactions with gephyrin, as the mobility of GlyRs outside gephyrin clusters (in control condition) was much higher and was similar to that seen with chronic blockade of GlyRs (Fig. 4*D–G*). Gephyrin interaction with the GlyRβ subunit has been suggested to decrease the lateral mobility of GlyRs (Schrader et al., 2004; Specht et al., 2011) through a phosphorylation-dependent mechanism (Zita et al., 2007; Charrier et al., 2010). And PKC-mediated phosphorylation enhanced postsynaptic localization of GABA_A_ receptors (Bannai et al., 2015). In the present study, treatment of the PKC inhibitor, but not the Ca^2+^ chelator, disturbed activation-dependent GlyR stabilization at postsynapses (Fig. 5*D*,*E*), suggesting that activation-dependent postsynaptic GlyR clustering in mature neurons may be mediated by the activity of Ca^2+^-independent PKC isoforms. Indeed, enhancement of glycinergic responses via PKC activation in hippocampal neurons (Schönrock and Bormann, 1995), and more specifically by Ca^2+^-independent PKC activity in sacral dorsal commissural nucleus, has been reported (Xu et al., 1996). In addition, ligand-induced conformational changes in GlyRs resulting from their activation may also mediate interactions with gephyrin (or associated proteins) and account for increased stability and cluster size (Meier, 2003). A structural analysis of GlyR using electron cryomicroscopy (Du et al., 2015) has recently reported that ligand binding induces dynamic changes in overall architecture, and the activation-induced conformational changes extend across both α- and β-subunits (Lynch, 2004). Indeed, such activation-dependent conformational changes affecting receptor clustering have been reported for the epidermal growth factor receptor (Ichinose et al., 2004), and a similar mechanism in ligand-gated neurotransmitter receptors was also recently reported for both AMPA receptors (Constals et al., 2015) and GABA_A_ receptors (Gouzer et al., 2014; Lévi et al., 2015). Therefore, activation-induced conformational changes might result in the synaptic clustering of GlyRs in mature neurons.

Although GlyR activation rapidly enhances the synaptic localization of the receptors, interestingly, strychnine-induced GlyR inactivation needs a longer time to increase mobile fractions of previously activated GlyRs at mature synapses (Fig. 5*B*,*C*). While the present observation seems inconsistent with the previous report demonstrating that strychnine application for 24 h decreases the density of previously clustered GlyRs in zebrafish Mauthner cells, this effect of strychnine on the GlyR density is reduced with animal development (Yamanaka et al., 2013). Indeed, Seitanidou et al. (1992) also reported that localizations of GlyRs are less affected by strychnine treatment in adult animals *in vivo*. These previous reports and our present study suggest that once glycine receptors are activated at postsynaptic sites, they can stay for longer durations in the mature stage. Although the stable localization of GlyR at synapse over 36 h seems longer than the half-life of surface GlyRs (Rasmussen et al., 2002), the previous study measured the turnover rate of nonspecific GlyR subtypes from all of the cell surface, including extrasynaptic GlyRs. Indeed, turnover rates of exocytosis/endocytosis in AMPA receptors and GABA_A_ receptors are higher in the extrasynaptic area than at synapses (Bogdanov et al., 2006; Opazo and Choquet, 2011), and this is thought to occur in glycine receptors (Rosenberg et al., 2001; Dumoulin et al., 2009). Thus, the long-lasting stability of GlyRs observed in the present study may suggest that the localization of α1-subunit-contained GlyRs is robust at synapses. Furthermore, the increased mobile fraction of GlyRs, which were treated with strychnine for 48 h, is comparable to the cSTR condition, indicating that the half-life of stabilized GlyRs at synapses is nearly 24 h. Although activation-dependent postsynaptic GlyR stabilization occurs even within 1 h, the destabilization of previously activated GlyR takes over 24 h. This discrepancy in the time required for increasing or decreasing postsynaptic stabilization may suggest that GlyR activation is more important for the formation of synaptic clustering of GlyR rather than for maintenance in mature neurons.

In spinal cord and brainstem, the dynamic shift of released inhibitory neurotransmitter contents occurs during development in presynapses (Nabekura et al., 2004). In parallel, postsynaptic modulation is necessary for the fine-tuning of inhibitory synaptic efficacy in mature neurons. It is known that predominant subunit composition of GlyRs developmentally shifts from homomeric GlyRα2 to heteromeric GlyRα1β (Lynch, 2004). Hence, the β-subunit of GlyRs that bind to gephyrin enables localization to synaptic sites (Tyagarajan and Fritschy, 2014). In addition to the developmental changes in GlyR subunit composition, the present study demonstrates that GlyR activation is critical for the postsynaptic clustering of the receptor. Therefore, the formation of appropriate inhibitory synapse in mature neurons occurs in both presynapses and postsynapses. And postsynaptic receptor distributions are constantly regulated by released neurotransmitters in an activation-dependent manner without Ca^2+^-mediated depolarizing responses in the mature neural system.

Together, activation-dependent synaptic clustering of GlyRs in mature neurons might be mediated by modulating the interactions between GlyRs and gephyrin via PKC-mediated signaling. Although the precise mechanisms of activation-dependent GlyR dynamics and gephyrin interactions remain to be fully elucidated, our data demonstrate that activation-dependent postsynaptic GlyR clustering rapidly occurs in mature neurons and may be involved with mechanisms different from those at developing synapses. The dynamic molecular changes we report here are likely to contribute to a postsynaptic mechanism underlying the developmental switching of inhibitory neurotransmission and the activity-dependent functional strengthening of inhibitory synaptic transmission in the adult CNS.

## Synthesis

Reviewing Editor: Vivian Budnik, University of Massachusetts Decisions are customarily a result of the Reviewing Editor and the peer reviewers coming together and discussing their recommendations until a consensus is reached. When revisions are invited, a fact-based synthesis statement explaining their decision and outlining what is needed to prepare a revision will be listed below. The following reviewer(s) agreed to reveal their identity: Michele Jacob.

Reviewers were satisfied with the response to the original reviewers and found the work significant. However, they felt that a few issues should be addressed. While most of these are issues for discussion or analysis of already existing data, there are also some experimental work that needs to be conducted before the paper can be considered for publication. These issues are summarized below.

Further Data Analysis

1. The authors should conduct further analysis of their mIPSCs data to include a kinetics analysis, as a developmental change in GlyR subunit composition has been reported to shorten these kinetics. Thus, it is important to determine if chronic GlyR composition has any impact in this process.

Discussion/text clarification

1. Based on an mIPSP inter-event-interval analysis, the authors suggest that chronic strychnine has little effect on presynaptic glycine release. However, the frequency of minis is also determined by the number of functional synapses. Thus, the authors should clarify that strychnine has little effect in both presynaptic release probability and the formation of active Gly synapses.

2. The interpretation of the FRAP experiments in the presence of the heteromeric GlyR blocker is unclear, as ectopically expressed SEP-GlyR1 are likely to also form homomeric GlyRs. This concern should be discussed.

3. Given the timing of manipulations, it is unclear if the data shown represents a physiological conditions. Authors should elaborate about this concern.

4. The conceptual advance of this study is reduced if the underlying mechanism is mediated by interactions between GlyR beta and gephyrin, as this process has been already characterized in previous publications. The authors should discuss this.

Experiments

1. A potential change in GlyR beta and/or alpha1 subunit phosphorylation going from chronic strychnine block to GlyR activation in mature cultures should be assessed.

2. 1 µM strychnine is known to also affect nicotinic AChRs, which function both pre- and postsynaptically. The authors should test if a potential alteration in nAChR function might influence the change in GlyR clusters. For example, the authors could determine if blocking nAChRs has any consequence on GlyR clusters, or if the strychnine concentration used does affect nAChR function in their system.

3. The recovery in FRAP experiments is difficult to see in Fig.4C. A potential concern is that photobleaching itself might alter mCherry gephyrin clusters. The authors should assess whether photobleaching affects the mCherry gephyrin clusters, puncta size and intensity.
